# Association of job stress, FK506 binding protein 51 (FKBP5) gene polymorphisms and their interaction with sleep disturbance

**DOI:** 10.7717/peerj.14794

**Published:** 2023-01-30

**Authors:** Peixin Li, Yuxi Wang, Baoying Liu, Chuancheng Wu, Chenzhou He, Xuejie Lv, Yu Jiang

**Affiliations:** Department of Preventive Medicine, Fujian Provincial Key Laboratory of Environment Factors and Cancer, Key Laboratory of Environment and Health, School of Public Health, Fujian Medical University, Fuzhou, China

**Keywords:** Job stress, Sleep disturbance, Gene–environment interaction (G×E), FKBP5

## Abstract

**Background:**

Sleep disturbance is an outcome of multiple factors including environmental and genetic influences. Job stress, a complex environmental factor, likely affects sleep quality, significantly reducing the quality of life of workers. Additionally, FK506 binding protein 51 (FKBP5) may be a pathogenic factor for sleep disturbance as it regulates hypothalamic–pituitary–adrenal (HPA) axis activity, where HPA axis has been found to be involved in the regulation mechanism of sleep and stress response.

**Objectives:**

The main aim of this study was to investigate the association between job stress and FKBP5 gene polymorphism as well as their interaction with sleep disturbance in Chinese workers; to date, these relationships have not been explored.

**Methods:**

This is a cross-sectional study. A total of 675 railway workers (53.8% male) completed a short Effort-Reward Imbalance questionnaire and the Pittsburgh Sleep Quality Index. The SNaPshot single nucleotide polymorphism (SNP) assay was carried out by screening for FKBP5 SNPs in every participant. Generalized multifactor dimensionality reduction (GMDR) was used to identify the strongest G×E interaction combination.

**Results:**

The findings showed that job stress was significantly associated with sleep disturbance; specifically, scores on the PSQI subscales (sleep disturbance, sleep medication, and daytime dysfunction) exhibited significant differences between the two job stress groups (X^2^ = 18.10, *p* = 0.01). Additionally, the FKBP5 SNP rs1360780-TT (adjusted odds ratio [AOR] = 4.98, 95% confidence interval [CI] = 2.80–8.84) and rs3800373-CC genotype (AOR = 2.06, CI = 1.10–3.86) were associated with an increased risk of sleep disturbance. Job stress and rs1360780 and rs3800373 variants showed a high-dimensional interaction with sleep disturbance as determined by the GMDR model.

**Conclusion:**

The FKBP5 gene may increase susceptibility to job stress and result in sleep disturbance, especially in the presence of negative work-related events. These findings contribute to the field of sleep disturbance prevention and treatment.

## Introduction

Sleep is a critical component of physical and emotional health and well-being, but sleep disturbance is widely prevalent. Studies have revealed that 22.6% of individuals aged 18–25 years experience frequent episodes of insomnia, and 11.3% of people aged 26–40 years and 15.7% of people aged 41–65 years have difficulty maintaining sleep, according to observations in the Netherlands, United Kingdom and United States (Kocevska et al., 2021). Sleep issues are most prevalent in those with paid work (Kocevska et al., 2021). A meta-analysis showed that the prevalence of insomnia in the general population of China reached 15.0% (Cao et al., 2017). Sleep-related health problems not only increase the susceptibility to neuropsychological diseases, but also increase the risks of metabolic diseases, neurodegenerative diseases, and cancer (Daskalakis & Binder, 2015; Duchaine et al., 2020). The negative effects of sleep disturbance lead to lower work capacity and more mistakes by workers; consequently, these workers pose health and safety risks, including accidents and injuries (Parkes, 2017).

It is well- known that environmental and genetic factors contribute to sleep disturbance (Kimura & Winkelmann, 2007). Some of the most influential environmental factors are work-related elements, including the imbalance between effort and reward, job stress, excessive working hours, and high-demand/low-control job training (Li et al., 2021). In the early 1990s, Siegrist introduced the effort-reward imbalance (ERI) model in the field of job stress epidemiology. This theory postulates that an imbalance between high “costs” (exerting great effort at work) and low benefits (salary, the possibility for promotion, and positive feedback) produces job stress that affects both mental and physical health (Theorell, 2017). Workers with higher levels of job stress or ERI are more likely to suffer from difficulty falling asleep or maintaining sleep (Ota et al., 2009; Davies et al., 2017). In addition, individuals who perceive high job stress are vulnerable to sleep disturbances, and those exposed to ERI have a higher risk of absence due to illness (Rugulies et al., 2009; Götz et al., 2018). Cho & Chen (2020) in a 4-year follow-up study, reported that ERI and sleep problems have a reciprocal relationship among older workers. These findings indicate that exploring the relationship between ERI and sleep disturbance may have important implications for sleep health.

Although many studies have identified contributing factors of sleep disturbance, the extent to which the interaction between genetic and environmental factors affects the manifestation of sleep disturbance is still unknown. A large UK twin study reported that the heritability of sleep quality was approximately 43% (Barclay et al., 2010). Several researchers have reported the effect of occupational stress on sleep quality and the role of the PER3 gene in sleep disorders (Peng et al., 2022). In addition, other studies have acknowledged that these factors work in concert to influence sleep *via* processes such as a gene × job stress interaction (Garfield, 2021; Peng et al., 2022). More interestingly, recent studies have shown interactions between some hypothalamic-pituitary-adrenal (HPA) axis-related gene (*e.g.*, 5-HTTLPR and CRHR1) polymorphisms and stress on sleep (Utge et al., 2010; Huang et al., 2014). The above studies confirm that genetic and environmental factors may interact and influence sleep and that HPA axis genes may play an important role in sleep disturbance.

The effects of these factors on sleep may be modulated by the ability to deal with individual stress, the HPA axis and the neuroendocrine stress response system; these modulators may be promising candidate gene sources to explore with a gene × environment interaction approach. FK506 binding protein 51 (FKBP5), a cochaperone of Hsp90 and a component of the chaperone-receptor heterocomplex, has been shown to promote the homeostatic regulation of the HPA axis through the inhibition of glucocorticoid receptor (GR) activity, which is the principal biological mechanism underlying the stress response (Hartmann et al., 2012). Indeed, a recent study provided evidence that FKBP5 mediates the associations among the HPA axis, GR and the development of sleep disturbance (Buckley, Duggal & Schatzberg, 2008). One of the key regulatory proteins of the GR receptor complex, FKBP5 is located on chromosome 6 p 21.31 and is considered a stress-related gene (Klengel et al., 2013). Moreover, FKBP5 not only plays an important role in regulating the HPA axis and stress response but also has a significant impact on sleep (Albu et al., 2014). FKBP5 single nucleotide polymorphisms (SNPs) (*i.e.,* rs3800373 and rs1360780) may increase suicide risk in individuals with a history of childhood trauma (Roy et al., 2010; Womersley et al., 2022). As many studies have reported, FKBP5 variants have been linked to neuropsychiatric conditions, including depression, anxiety, posttraumatic stress disorder and cognitive impairment, all of which share some symptoms with sleep disturbance (Wang, Shelton & Dwivedi, 2018; Hernández-Díaz et al., 2019; Terrelonge et al., 2022). In addition, a study reported a potential association between the FKBP5 rs9470080 variant and subjective health complaints, in which the FKBP5 rs9470080 CC genotype was associated with poor sleep quality complaints in the female working population (Sannes et al., 2020). Since rs1360780, rs3800373 and rs9470080 are likely functional variants, we decided to focus on these particular SNPs.

Recent studies have shown that FKBP51 mRNA is widely expressed in the hypothalamus and brainstem, which are brain regions important for sleep-wake regulation (Scharf et al., 2011). As FKBP5 is involved in the regulation of HPA activity, which is known to influence sleep, we were interested in determining whether FKBP5 gene variants affect the risk of sleep disturbance. However, research assessing the interplay between FKBP5 gene polymorphisms and job stress as well as their impact on sleep disturbance is scarce. G×E interaction research provides a potential pathway for understanding how genetic differences influence the likelihood that exposure to job stress will result in sleep disturbance.

Therefore, the aims of the present study were to investigate the effects of FKBP5 gene polymorphisms and job stress on sleep disturbance in Chinese workers. Furthermore, we explored the interaction effects through the higher-order GMDR model.

## Materials & Methods

### Study population

This study was part of the Occupational Health Study of Railway Workers (OHSRW), carried out from October 2019 to May 2020. A detailed questionnaire was used to collect sociodemographic and lifestyle information, accompanied by measurements of sleep disturbance and job stress during the annual occupational health examination. Blood samples were taken from every participant between 7 and 9 AM on the same day as part of the health examination. The inclusion and exclusion criteria and control of confounding factors have been previously described (Wang et al., 2022). Specifically, participants were included who had been working in this particular job (front-line railway) for >1 year and were aged between 20 and 60 years. The present study included 690 participants who consented, of whom 15 participants were excluded due to inadequate information or missing blood samples; thus, 675 participants (363 males and 312 females) from the China Railway Fuzhou Branch were included in the final analysis. This study was approved by the Ethics Committee of Fujian Medical University (No. 2019025). Written informed consent was obtained from each participant.

### Job stress

The Effort-Reward Imbalance (ERI) questionnaire is one of the most widely used instruments to estimate job stress, and it is based on Siegrist’s Effort-Reward Imbalance (ERI) model (Siegrist et al., 2009). The job stress test has been previously described (Wang et al., 2022). Specifically, an ERI ratio >1 represents an imbalance between effort and reward, which is considered job stress (Wu et al., 2019). In the present study, Cronbach’s alpha of this questionnaire was 0.882.

### Sleep disturbance

The Pittsburgh Sleep Quality Index (PSQI) is a 19-item self-report questionnaire designed to evaluate sleep quality during a one-month interval (Buysse et al., 1989). The PSQI has high internal consistency, reliability and construct validity and consists of seven clinically derived components. Each dimension is scored from 0 to 3; total PSQI scores range from 0 to 21. Participants with a total score higher than 5 were classified as having sleep disturbance (Carpenter & Andrykowski, 1998).

### Genotyping

A 5-ml fasting venous blood sample was collected from every participant between 07:00 and 09:00 at the workplace. According to the relevant references (Roy et al., 2010; Fudalej et al., 2015; Sannes et al., 2020; Womersley et al., 2022), the genotype information of SNPs in the Chinese Human Genome (CHB) and the gold standard (*i.e.,* *r*2 = 0.8, MAF>15% standard), the HapMap database (http://hapmap.ncbi.nlm.nih.gov/) and Haploview software (http://www.broad.mit.edu/mpg) were used to select target tag SNP loci in this study (Wang et al., 2022). Genomic DNA was isolated and purified from the samples using a whole blood genome extraction kit (Beijing Think out Sci-Tech Co., Ltd). Selected FKBP5 SNPs (rs1360780, rs3800373 and rs9470080) were genotyped using SNaPshot analysis (Mehta et al., 2017). Table 1 shows the sequences of the primers.

**Table 1:** Primer information.

Primer	Direction	Sequence 5′-3′
rs1360780 _F	Forward	GGCATGGGCACTCTGAAAAGAT
rs1360780 _R	Reverse	TCTCTTGTGCCAGCAGTAGCAAGT
rs3800373 _F	Forward	GGCATGGGAAGCTGTCTTCAAC
rs3800373 _R	Reverse	CCAGCATTGCTACTGCTCAGCTTC
rs9470080 _F	Forward	TCTTTTCCAGGCTATGAATTGACAAA
rs9470080 _R	Reverse	TGTGTCCAGCCATGTGCTTTTT

### Statistical analysis

Statistical analyses were carried out using IBM SPSS version 26.0 (SPSS Inc., Chicago, IL, USA). Chi-square tests were used to compare sociodemographic characteristics. The chi-square goodness-of-fit test was used to determine the Hardy-Weinberg equilibrium (HWE) of the FKBP5 gene rs1360780, rs3800373 and rs9470080 polymorphisms in our sample. The Mann–Whitney U test was used to assess the relationship between job stress and sleep disturbance. Unconditional logistic regression was used to evaluate the relationship of genotypes and job stress with the risk of sleep disturbance, after adjusting for sex, age, ethnicity, marital status, smoking status, and alcohol consumption. Bonferroni correction was applied as a multiplicity correction. Generalized multifactor dimensionality reduction (GMDR, http://sourceforge.net/projects/gmdr/) was used to screen the strongest G×E interaction combination (Lou, 2015). We conducted 10-fold cross-validation (CV) to avoid unstable results and obtained a robust averaged result. All reported *P* values are two-tailed, and those less than 0.05 were considered statistically significant.

## Results

### Sociodemographic characteristics of participants

The demographic characteristics and sleep disturbances of participants are summarized in Table 2. In this study, 363 males (53.8%) and 312 females (46.2%) were included. There were significant differences in the distribution of sleep disturbances between different job stress groups (*x*^2^ = 18.10, *p* = 0.01). We found that there were no significant differences in demographic characteristics between the people that did not report sleep disturbance and sleep disturbance groups (*p* > 0.05).

**Table 2:** Demographic characteristics of job stress and sleep disturbance (*n* = 675).

Variables	N	Non-sleep disturbance (%)	Sleep disturbance (%)	*χ* ^2^	*p*-value
Gender					
Male	363	221 (60.9)	142 (39.1)	0.57	0.45
Female	312	181 (58.0)	131 (42.0)		
Age (years)					
≤ 30	160	95 (59.4)	65 (40.6)	3.68	0.30
31–40	236	147 (62.3)	89 (37.7)		
41–50	200	109 (54.5)	91 (45.5)		
>51	79	51 (64.6)	28 (35.4)		
Ethnicity					
Han	530	318 (60.0)	212 (40.0)	0.20	0.65
Minority	145	84 (57.9)	61 (42.1)		
Marital status					
Unmarried	119	68 (57.1)	51 (42.9)	1.97	0.37
Married	520	316 (60.8)	204 (39.2)		
Divorced or widowed	36	18 (50.0)	18 (50.0)		
Smoking status					
Non-smoker	263	156 (59.3)	107 (40.7)	0.01	0.92
Smoker	412	246 (59.7)	166 (40.3)		
Alcohol consumption					
Non-drinker	362	219 (60.5)	143 (39.5)	0.29	0.60
Drinker	313	183 (58.5)	130 (41.5)		
Job stress					
Non-job stress	366	245 (60.9)	121 (44.3)	18.10	0.01
Job stress	309	157 (39.1)	152 (55.7)		

### Association between job stress and sleep disturbance

The PSQI total and subscale scores were compared between the two job stress groups using the Mann–Whitney U test. Significant differences in the PSQI (sleep disturbance, sleep medication, and daytime dysfunction subscales and total) scores were found between the two job stress groups (*p* = 0.019, *p* = 0.001, *p* = 0.005, and *p* = 0.003, respectively), as shown in Table 3. Moreover, after adjusting for confounding factors, the logistic regression investigating job stress and sleep disturbance showed that participants with job stress (ERI ratio >1) had a higher risk of sleep disturbance (OR = 1.98, 95% CI = 1.45−2.71).

**Table 3:** Association between the job stress and sleep disturbance and its subscale scores (*n* = 675).

	Job stress, mean (SD)		
Sleep disturbance	ERI ≤1	ERI>1	*P*-values
Subjective sleep quality	0.44 (0.75)	0.49 (0.80)	0.521
Sleep latency	0.86 (0.88)	0.81 (0.65)	0.919
Sleep duration	0.16 (0.42)	0.19 (0.51)	0.915
Sleep efficiency	0.68 (0.94)	0.59 (0.92)	0.117
Sleep disturbance	0.66 (0.66)	0.64 (0.84)	0.019
Sleep medication	0.43 (0.68)	0.77 (0.66)	0.001
Daytime dysfunction	0.63 (0.65)	0.80 (0.74)	0.005
PSQI total scores	3.86 (2.56)	4.29 (2.49)	0.003

**Notes.**

*P*-values for the two-groups comparison were determined by the Mann–Whitney U test.

### Relationships of three SNPs of the FKBP5 gene with sleep disturbance

All genotypes in the control group were distributed according to HWE (*p* > 0.05). For information about linkage disequilibrium between these SNPs, see Fig. S1. The rs1360780-TT genotype frequency was 18.3% in the sleep disturbance group and 4.7% in the non-sleep disturbance group. The rs3800373-CC genotype frequency was 9.5% in the sleep disturbance group and 4.7% in the non-sleep disturbance group. As shown in Table 4, The rs1360780-TT genotypes and job stress were associated with increased sleep disturbance risk (OR = 4.98, 95% CI = 2.80−8.84, *p* < 0.001, Bonferroni corrected *p* < 0.01); this relationship remained significant after controlling for covariates (AOR = 2.06, 95% CI = 1.10−3.86, *p* < 0.001, Bonferroni corrected *p* < 0.01). We also found no significant correlation between rs3800373 (after Bonferroni correction) or rs9470080 and susceptibility to sleep disturbance.

### Interaction between job stress and FKBP5 gene SNPs on sleep disturbance

**Figure 1:**
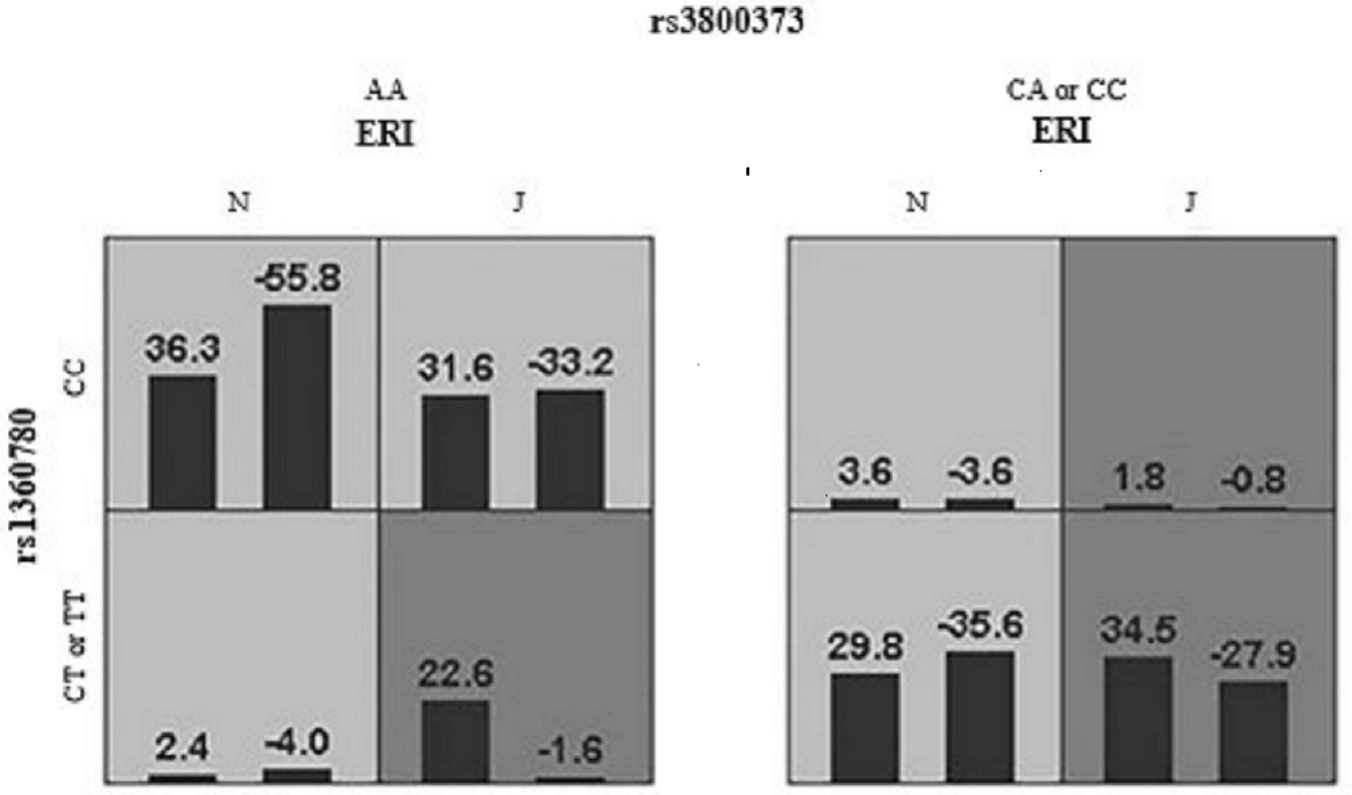
The GMDR results of the interaction between FKBP5 gene and job stress. High- and low- risk factors are inherently determined by the model, in which the dark gray box represents the high-risk factors, and the light gray represents the low-risk factors. Bars represent the maximum likelihood estimation of case weights. In the same box. the left column is positive score and the right column is negative score. N and J denote normal and job stress (ERI>1), respectively. Among them, individuals with job stress, CA or CC genotype of rs3800373 and CT or TT genotype of rs1360780 had the highest sum score.

Gene × environment interaction models were determined by GMDR analysis. As shown in Table 5, the strongest model of the three factors was the interaction between rs1360780 and rs3800373 and job stress, with a CV consistency of 10/10 and testing accuracy of 0.589 (*P* value = 0.011). Figure 1 shows the details of the results. We found that participants with job stress, the CA or CC genotype of rs3800373 and the CT or TT genotype of rs1360780 had the highest risk of sleep disturbances.

**Table 4:** Association analysis for 3 target SNPs of FKBP5 gene with sleep disturbance.

SNPs	Genotypes and alleles	Frequencies N (%)		OR (95%CI)^a^	*P*-values
		Non-sleep disturbance (*n* = 402)	Sleep disturbance (*n* = 273)		
rs1360780					
	CC	231 (57.5)	123 (45.1)	Ref	
	CT	152 (37.8)	100 (36.6)	1.24 (0.86–1.73)	
	TT	19 (4.7)	50 (18.3)	4.98 (2.80-8.84)^**^	
	C allele	614 (76.4)	346 (63.4)	Ref	
	T allele	190 (23.6)	200 (36.6)	1.88 (1.48-2.39)^**^	
HWE test for controls					0.811
rs3800373					
	AA	234 (58.2)	156 (57.1)	Ref	
	CA	149 (37.1)	91 (33.3)	0.92 (0.66–1.24)	
	CC	19 (4.7)	26 (9.5)	2.06 (1.10-3.86)^*^	
	A allele	617 (76.7)	403 (73.8)	Ref	
	C allele	187 (23.3)	143 (26.2)	1.18 (0.92–1.52)	
HWE test for controls					0.855
rs9470080					
	CC	187 (46.5)	140 (51.3)	Ref	
	CT	170 (42.3)	112 (41.0)	0.88 (0.64–1.22)	
	TT	45 (11.2)	21 (7.7)	0.63 (0.36–1.12)	
	C allele	544 (67.7)	392 (71.8)	Ref	
	T allele	260 (32.3)	154 (28.2)	0.83 (0.66–1.06)	
HWE test for controls					0.891

**Notes.**

a Adjusted for sex, age, ethnicity, marital status, smoking status, and alcohol consumption.

* *P* < 0.05.

** *P* < 0.001.

**Table 5:** Strongest gene-environment interaction models, as identified by GMDR.

Model	Testing accuracy (%)	Training accuracy (%)	*P*-value	Cross-validation consistency
ERI	0.583	0.585	*p* = 0.011	10/10
ERI ×rs1360780	0.585	0.575	*p* = 0.055	10/10
ERI ×rs1360780×rs3800373	0.589	0.585	*p* = 0.011	10/10

**Notes.**

Adjusted for gender, age, ethnicity, marital status, smoking status and alcohol consumption.

## Discussion

The present study examined the interaction between FKBP5 gene polymorphisms and job stress on sleep disturbances in a sample of Chinese workers. Job stress was significantly associated with sleep disturbance, as indicated by significant differences between the PSQI total score and its subscale (sleep disturbance, sleep medication and daytime dysfunction) scores between the two job stress groups. Moreover, the FKBP5 SNP rs1360780-TT genotypes was related to an increased risk of sleep disturbances. In the GMDR model, job stress, rs1360780 and rs3800373 showed a high-level interaction that influenced sleep disturbance.

Most epidemiological data have shown that job stress puts individuals at a high risk for sleep disturbance (Linton et al., 2015). In the present study, we observed that 40.4% of participants reported sleep disturbance, indicating that sleep disturbance among Chinese workers may be a serious public health problem. We found that job stress was a significantly associated with sleep disturbance and that job stress was an independent risk factor for sleep disturbance. These effects of job stress on sleep disturbance might be associated with physiological arousal due to activation of the HPA axis in stressful environments (Buckley & Schatzberg, 2005). When excessive job stress exceeds the body’s ability to cope, the imbalance affects the sleep of worker sleep, resulting in insomnia or sleep disturbance (Halonen et al., 2017). Moreover, long-term job stress increases adrenal sensitivity to adrenocorticotropic hormones, cortisol levels, and changes in glucocorticoid and growth hormone levels on the HPA axis, which may lead to inhibition of sleep and sleep disturbance (Matenchuk, Mandhane & Kozyrskyj, 2020; Asarnow, 2020).

Dysregulation of FKBP5 gene may disrupt the feedback loop of the HPA axis, ultimately leading to disruption of HPA axis homeostasis (Albu et al., 2014). Moreover, most stress-related hormones are known to promote wakefulness, elevated HPA activity appears to contribute to stress-induced sleep disorder (Buckley & Schatzberg, 2005). We observed an association between the FKBP5 genotype and sleep disturbance. In our study, participants with the TT genotype of rs1360780 had an increased risk of sleep disturbance after adjusting for confounding factors. Indeed, FKBP5 SNPs were previously found to be associated with affective disorders such as those arising from maladaptation to stress (De Kloet, Joëls & Holsboer, 2005), with diagnosed patients displaying altered sleep patterns (Steiger & Kimura, 2010). Mice lacking the gene encoding FKBP5 (51KO mice) demonstrated more active stress-coping behavior and improved sleep profiles (Hartmann et al., 2012). In contrast, individuals carrying the T allele of rs1360780 who were exposed to early-life stress had a higher risk of posttraumatic stress disorder; thus, the interaction between these genes may increase the likelihood of developing stress-related disorders (Wang, Shelton & Dwivedi, 2018).

Interestingly, we found significant G×E interactions by performing GMDR. GMDR is an emerging method of G×E analysis that reduces the false positive rate and improve accuracy through cross validation and permutation tests (Lou et al., 2008). Using GMDR, we found that individuals carrying the CA/CC genotype (rs3800373) and CT/TT genotype (rs1360780) were at greatest risk of sleep disturbance, when experiencing job stress. Thus, FKBP5 polymorphisms may affect sleep disturbance through interactions with job stress. Our results suggest that environmental and genetic factors interact to influence sleep disturbance, as reported in some studies (Li et al., 2021; Garfield, 2021). Gene × environment interaction research has been primarily guided by the diathesis-stress model (Belsky & Pluess, 2009; Belsky & Hartman, 2014) which establishes that individuals carrying “vulnerable genes” are more susceptible to the effect of environmental adversity and thus more prone to developing psychological or behavioral problems (Monroe & Simons, 1991; Belsky & Pluess, 2013; He et al., 2019). In this study, the CT/TT genotype in rs1360780 of FKBP5 are probably the genetic variants or polymorphisms that make individuals vulnerable to stressful environments. Furthermore, the FKBP5 rs3800373 variant is located in the 3′ prime untranslated region and likely alters the stability and half-life of the mRNA and modulates glucocorticoid signaling and HPA axis function (Fudalej et al., 2015); this variant has been linked to symptoms directly associated with sleep disturbances such as anxiety, depression and pain (Knisely et al., 2019). On the other hand, previous studies have reported that the rs9470080 CC genotype was linked to an increased risk of low diurnal cortisol levels and likely leads to inattention, irritability and sleep problems through dysregulation of the HPA axis and FKBP5 (Isaksson et al., 2015). However, our study found no significant correlation of rs9470080 or rs3800373 (after Bonferroni correction) with susceptibility to sleep disturbance. The role of these two SNPs in job stress and sleep disturbance needs to be investigated in future studies.

To the best of our knowledge, this is the first study to analyze the high-dimensional interaction among job stress and FKBP5 genetic variants or polymorphisms on sleep disturbance. However, this study has several limitations. First, all of the subjects were railway workers, and occupational peculiarities may result in a lack of external validity due to selection bias. Second, we could not draw any definitive causal conclusions about relationships among job stress, the FKBP5 gene, and sleep disturbance or their interaction since this study used a cross-sectional design. Third, the limited sample size (*n* = 675) may lead to false-positive or false-negative results due to the lack of statistical power. Therefore, our current findings need to be validated in studies with larger sample sizes before any firm conclusions can be drawn. Finally, the PSQI scores were used to evaluate sleep disturbance; thus, the study still lacks objectivity compared with common diagnostic methods. Therefore, future studies should include objective measurement methods (such as actigraphy and objective PSG indices), use an extended sample size, take people in other professions as subjects and explore how these particular polymorphisms affect HPA axis homeostasis to provide better sleep quality profiles.

## Conclusions

In summary, our results showed that chronic exposure to job stress tends to be associated with sleep disturbance and that FKBP5 rs1360780 gene polymorphisms were associated with sleep disturbance. More importantly, these two polymorphisms and job stress interacted to affect sleep disturbance, indicating that the impact of job stress on sleep disturbance may be regulated by genotype. Our results suggest that the CA or CC genotype of rs3800373 and the CT or TT genotype of rs1360780 may be stress-responsive risk genotypes, supporting the diathesis-stress model.

##  Supplemental Information

10.7717/peerj.14794/supp-1 Data S1 Raw data

10.7717/peerj.14794/supp-2 Supplemental Information 2 Data introduction

10.7717/peerj.14794/supp-3 Figure S1 The linkage disequilibrium heatmap between the SNPs was measured using *r*^2^ and the absolute value of D’ The linkage disequilibrium heatmap between the SNPs was measured using r2 and the absolute value of D’. The relative positions of FKBP5 SNPs and the numbers in the squares refer to pair-wise linkage disequilibrium.

10.7717/peerj.14794/supp-4 Supplemental Information 4 Test Instrument Permissions

10.7717/peerj.14794/supp-5 Supplemental Information 5 ERI original questionnaire

## Additional Information and Declarations

### Data Availability

The following information was supplied regarding data availability: The raw measurements are available in the Supplemental Files.
