# Supplementary material for: Association of job stress, FK506 binding protein 51 (FKBP5) gene polymorphisms and their interaction with sleep disturbance
Source: PeerJ. 2023 Jan 30;11:e14794. doi: 10.7717/peerj.14794 (PMC9893914; doi:10.7717/peerj.14794)
Supplement: Supplemental Information 2 [file peerj-11-14794-s002.docx]

**Data-introduction**

Gender: male=1, female=2

Age: ≤30=1, 31-40=2, 41-50=3, ＞51=4

Ethnicity: Han=1, Minority=2

Marital status: Unmarried=1, Married=2, Divorced or Widowed=3

Smoking status: Non-smoker=1, Smoker=2

Alcohol consumption: Non-drinker=1, Drinker=2

ERI group: ERI≤1=1, ERI>1=2

PSQI group: Normal=0, Sleep Disturbance=1

rs1360780a: CC=1, CT=2, TT=3

rs3800373a: AA=1, CA=2, CC=3

rs9470080a: CC=1, CT=2, TT=3

rs1360780b: CC=1, CT or TT=2

rs3800373b: AA=1, CA or CC=2
