# Supplementary figures and images for: Association of job stress, FK506 binding protein 51 (FKBP5) gene polymorphisms and their interaction with sleep disturbance
Source: PeerJ. 2023 Jan 30;11:e14794. doi: 10.7717/peerj.14794 (PMC9893914; doi:10.7717/peerj.14794)

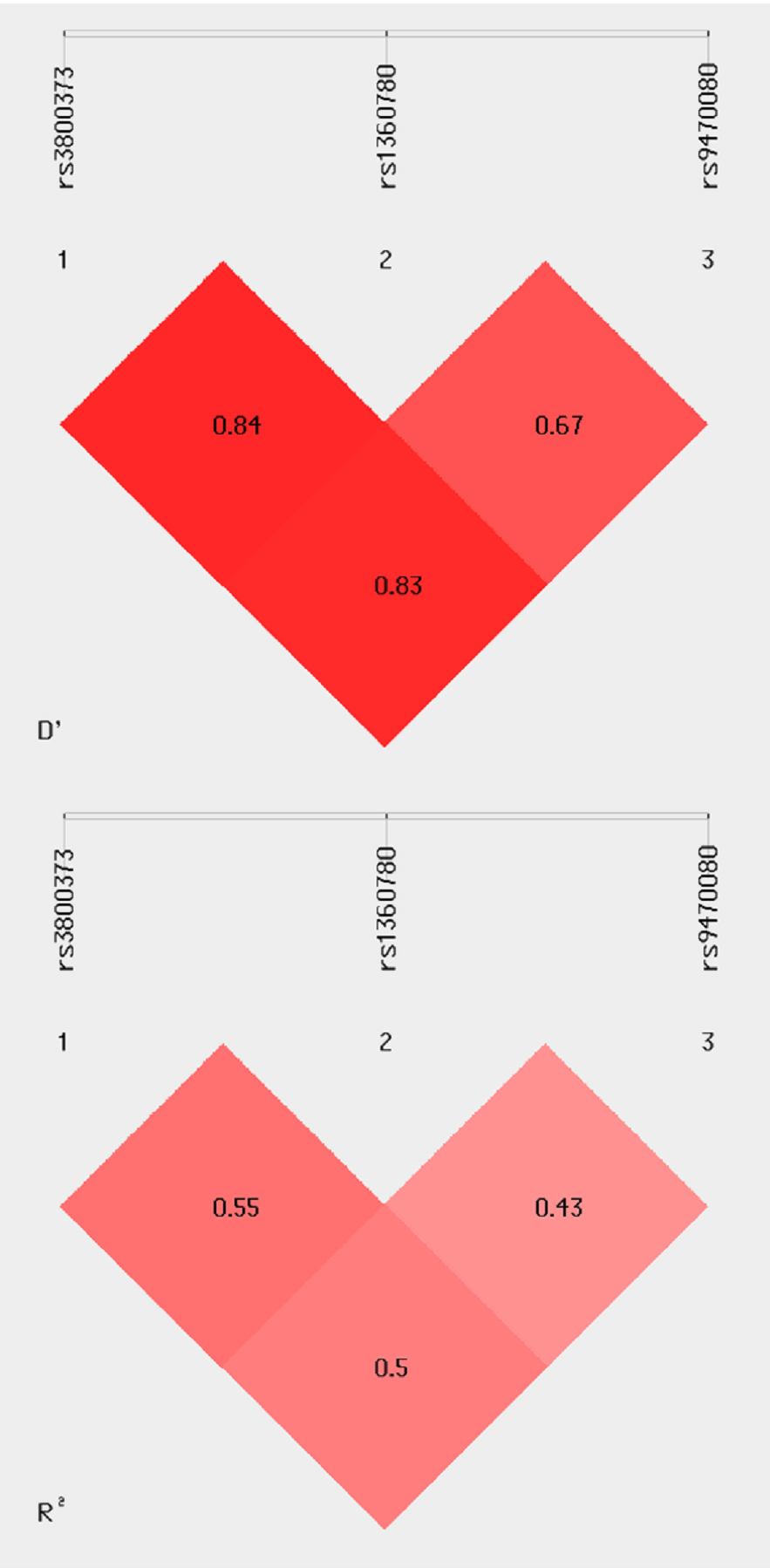

Supplement: Figure S1 — The linkage disequilibrium heatmap between the SNPs was measured using r2 and the absolute value of D’. The relative positions of FKBP5 SNPs and the numbers in the squares refer to pair-wise linkage disequilibrium. [file peerj-11-14794-s003.png]

ERI original questionnaire


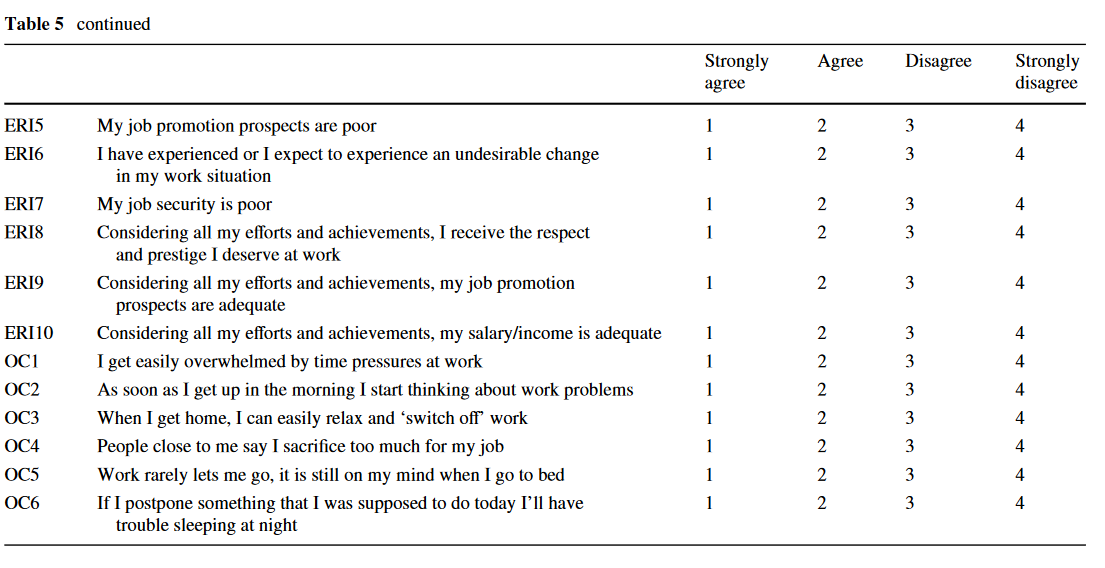

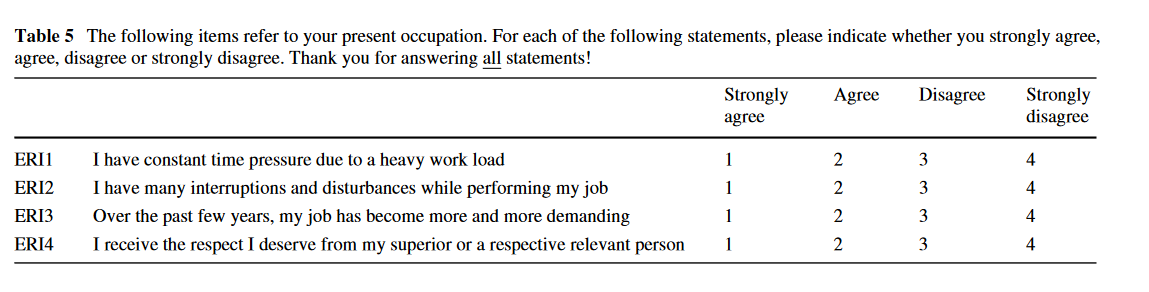

Supplement: Supplemental Information 5 [file peerj-11-14794-s005.docx]
