# Supplementary material for: Association of job stress, FK506 binding protein 51 (FKBP5) gene polymorphisms and their interaction with sleep disturbance
Source: PeerJ. 2023 Jan 30;11:e14794. doi: 10.7717/peerj.14794 (PMC9893914; doi:10.7717/peerj.14794)

Test Instrument Permissions

We have translated an existing questionnaire. The details of the original questionnaire are attached to the openly published articles, which our study makes cited in the methods section and in the references. The original questionnaire is attached here.
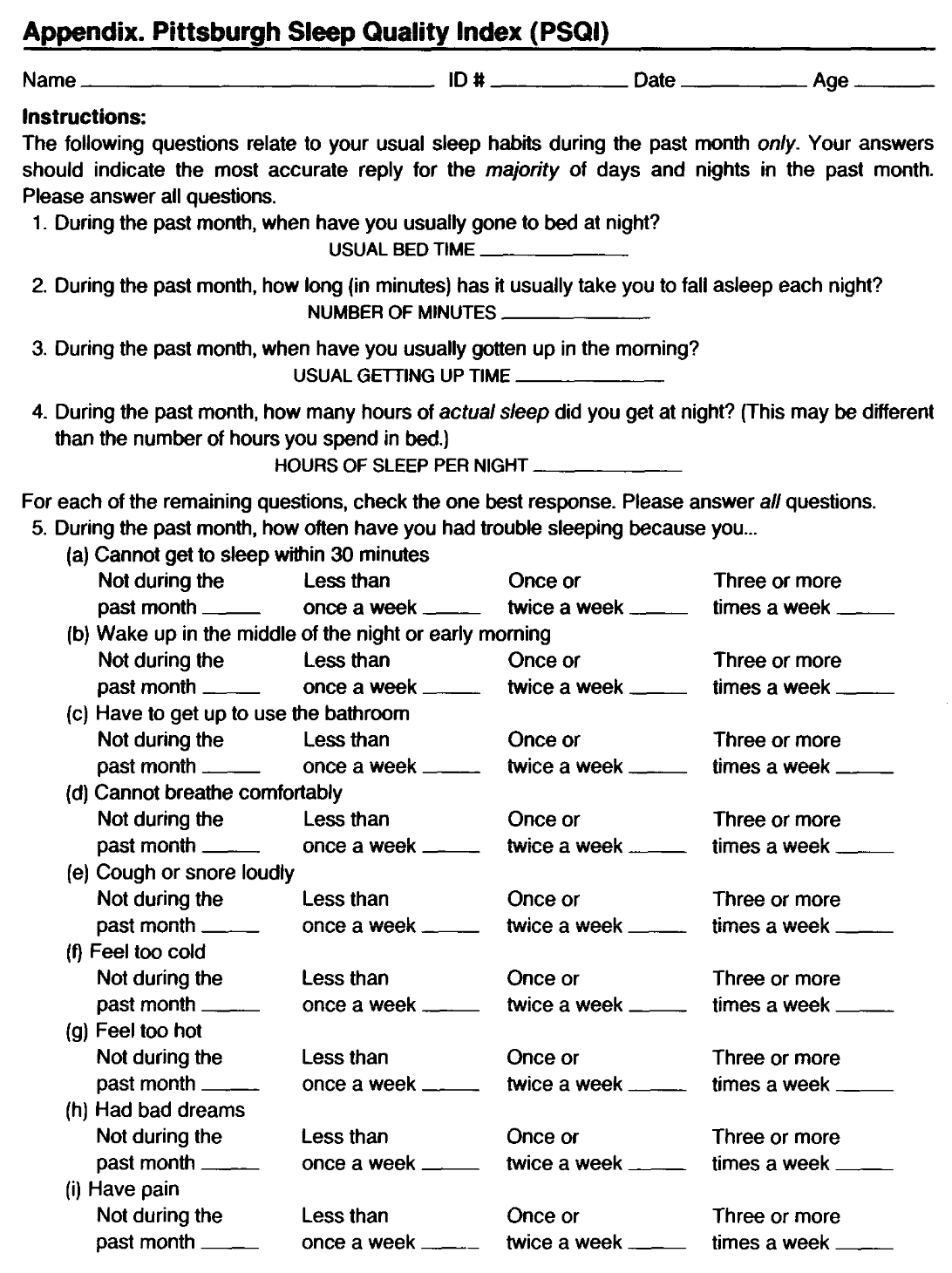

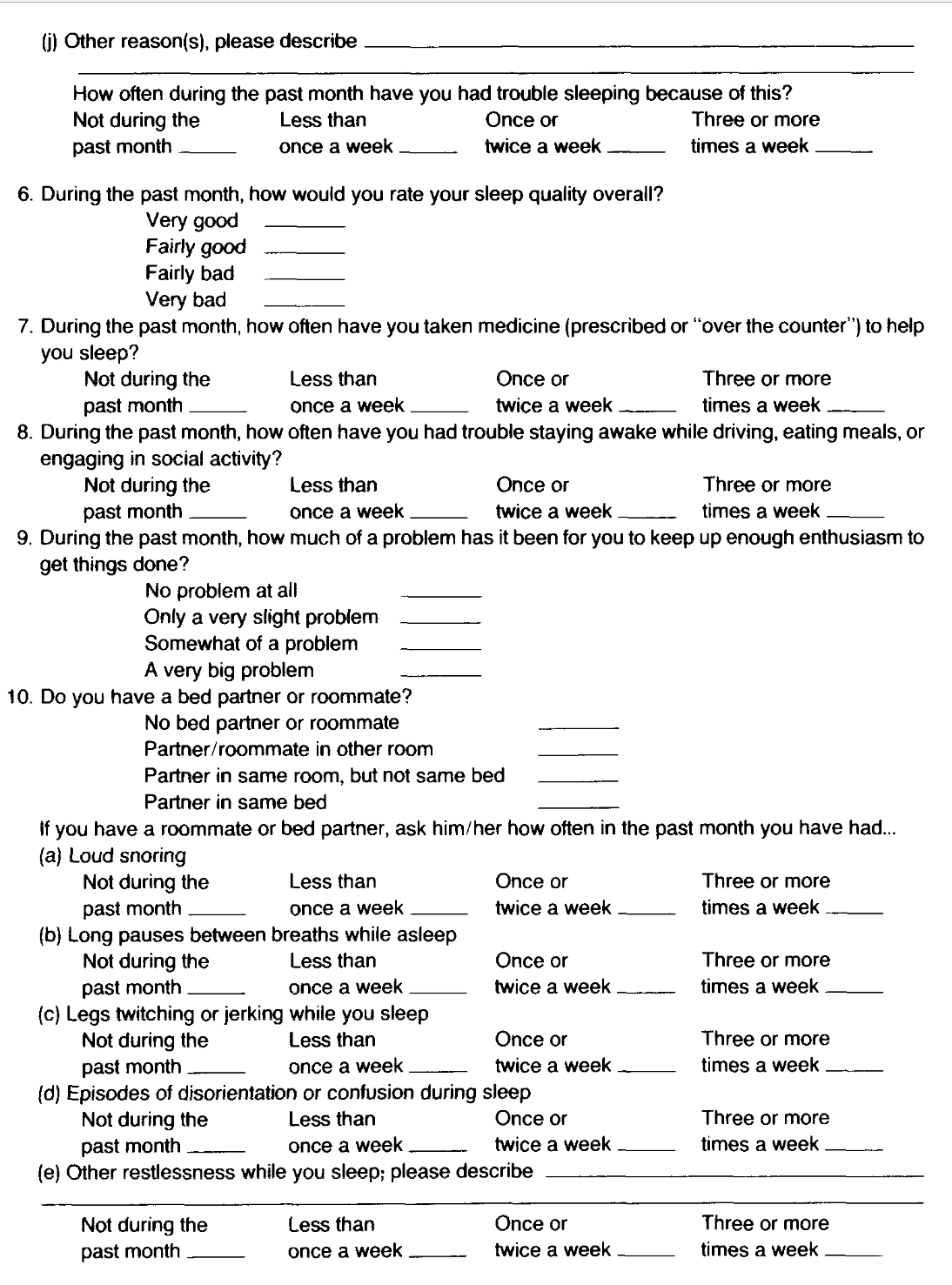


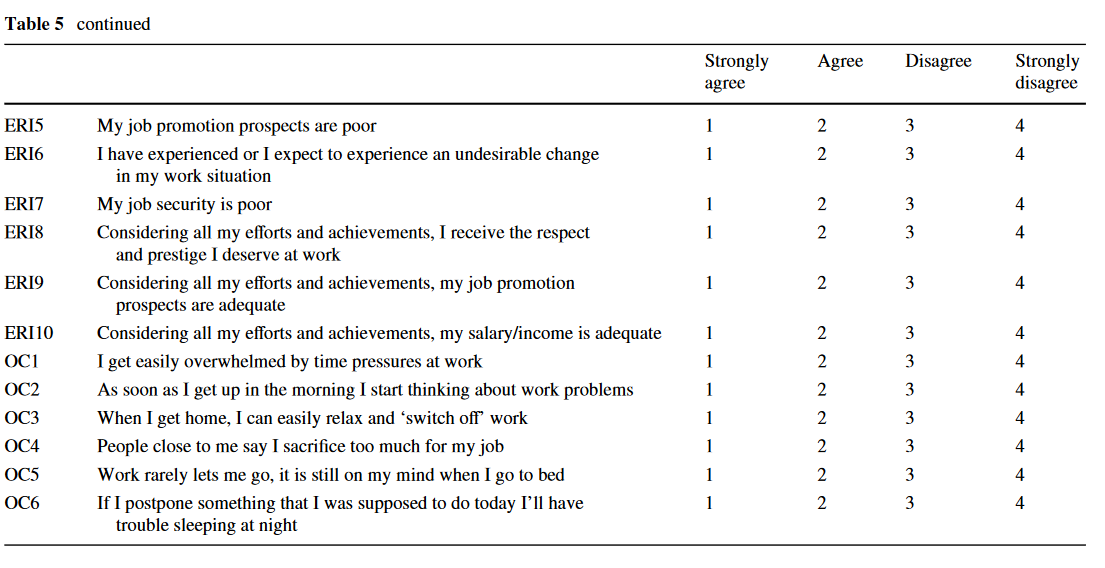

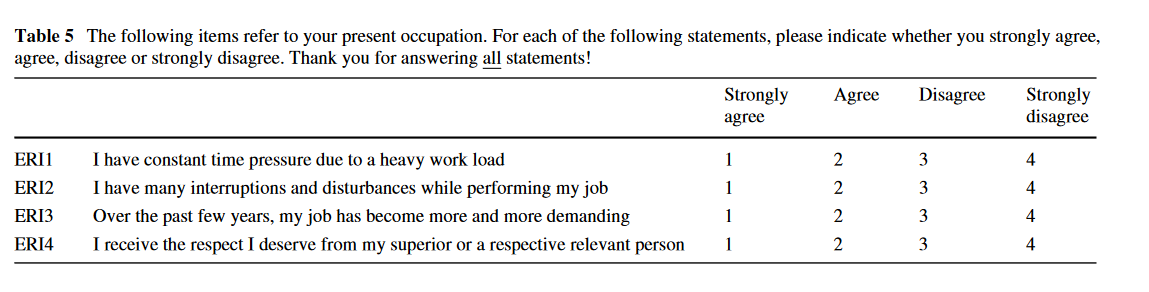

Supplement: Supplemental Information 4 [file peerj-11-14794-s004.docx]
